# Lutein Alleviate Acute Lung Injury Induced by Limb Ischemia-Reperfusion Through PPAR-γ/PI3K/AKT/NLRP3 Signaling

**DOI:** 10.1155/mi/2371545

**Published:** 2025-11-17

**Authors:** Chao Nie, Zhen Liu, Liang Zhang, Chuanchuan Liu, Hui Jiang, Minghua Liu

**Affiliations:** Department of Emergency, The First affiliated Hospital, Army Medical University, Chongqing, China

**Keywords:** acute lung injury, inflammatory response, ischemia reperfusion, lutein, pyroptosis

## Abstract

The detachment of cardiogenic emboli, the formation of arterial thrombosis, and the use of tourniquets in emergency situations of massive hemorrhage can all lead to acute limb ischemia. In order to save the limb, blood flow must be rapidly restored. However, the resulting ischemia reperfusion (IR) injury may trigger systemic inflammatory responses of varying degrees, and cause damage to distant organs. Acute lung injury (ALI) caused by acute limb IR (LIR) can significantly affect the prognosis of patients. Despite a large number of previous studies, there is currently no specific exists for this condition. This study aims to investigate the potential benefits of lutein on ALI caused by LIR (LIR–ALI). Network pharmacology analysis was used to predict the key targets and signaling pathways of lutein in the treatment of ALI. In addition, we established a mice model of LIR–ALI. Histological, ELISA, and Western blotting analyses were performed to determine the therapeutic effects and potential mechanisms of action. Our study found that lutein dose-dependently mitigated lung oxidative stress, inflammatory cell infiltration, and pyroptosis-related protein expression induced by LIR. The protective effect is partially mediated by regulating the PPAR-γ/PI3K-AKT/NLRP3 pathway to inhibit pyroptosis.

## 1. Introduction

As the most widely used and effective first aid device for controlling bleeding in injured limbs during trauma incidents, tourniquets have been extensively applied in various trauma events [1]. In addition, limb replantation, thrombosis, or other medical accidents can cause limb ischemia [2]. When oxygenated blood is reintroduced into the ischemic tissue, skeletal muscle ischemia reperfusion (IR) injury occurs. There is ample evidence indicating that prolonged skeletal muscle IR can trigger a systemic inflammatory response, leading to damage to distant organs. Among them, the lung, as the most vulnerable distant organ, is highly susceptible to acute lung injury (ALI) caused by limb IR (LIR–ALI) [3]. This may account for the high incidence of complications and mortality following orthopedic surgeries or prolonged use of tourniquets [4, 5].

In skeletal muscle with IR, inflammatory cytokines, oxygen-free radicals, and endogenous danger molecules can be released, and the complement system can be activated [6, 7]. In addition, endothelial cell damage caused by the activation of macrophages and neutrophils is one of the major pathological disorders of lung injury [8–11]. The lung's abundant blood supply and expansive pulmonary vascular surface area facilitate the accumulation of inflammatory factors and cells in the alveoli and interstitium, potentially leading to acute respiratory distress syndrome (ARDS) [12, 13].

Recent studies show that stimuli trigger danger-associated and pathogen-associated molecular patterns (DAMPs and PAMPs), inducing pyroptosis in alveolar epithelial cells and pulmonary macrophages, resulting in significant lung injury [14–16]. Pyroptosis is an inflammatory programmed cell death distinct from apoptosis and necrosis, marked by cell swelling, membrane rupture, and release of cytoplasmic contents. Pyroptosis is characterized by inflammasome activation triggered by various pathological stimuli. The NLRP3 inflammasome, a key indicator of pyroptosis, consists of NLRP3, active caspase-1, and ASC, an apoptosis-associated speck-like protein with a caspase activation and recruitment domain [17, 18]. The inflammasome is essential in the inflammatory response and is a key pathway for pyroptosis. The NLRP3-mediated classical pyroptosis pathway has the capacity to regulate and control pulmonary inflammation.

Lutein exhibits a range of pharmacological activities, such as antidiabetic, antioxidant, antiapoptotic, and anti-inflammatory effects, along with neuroprotective, renoprotective, and hepatoprotective properties [19–21]. Research indicates that lutein can mitigate IR injury in both the brain and skeletal muscles. However, it is still unclear whether lutein has a cytoprotective effect in ALI. Therefore, we speculate that lutein has a specific role in preventing diseases related to edema and inflammation.

## 2. Materials and Methods

### 2.1. Animals

**Figure 1:**
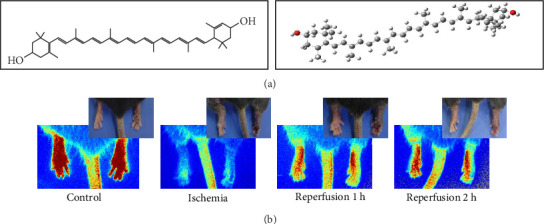
Chemical structure of lutein and the process of ischemia and reperfusion. (a) The chemical structure formula of lutein. (b) Blood perfusion at different times, measured by laser speckle analyzer.

The animal studies were conducted in accordance with the regulations approved by the Ethics Committee of Army Medical University (Approval Number: AMUWEC20242029). 30 male mice were randomly assigned to five groups (*n* = 6 each) via a random number table: (A) control group, (B) IR group, (C) IR + LLu group, (D) IR + HLu group, (E) IR + Lu+ GW9662. On the day of the experiment, lutein (C_40_H_56_O_2_; HPLC ≥ 90%) purchased from Solarbio (Beijing, China) was freshly prepared in corn oil. In accordance with previously reported doses [22, 23], the mice in the IR + HLu and IR + Lu+ GW9662 groups received lutein at a concentration of 25 mg/kg/day via gavage for five consecutive days, followed by IR 24 h after the final lutein administration. The IR + LLu group received lutein at a concentration of 10 mg/kg/day. Mice in the IR + Lu+GW9662 group were administered intraperitoneal injections of 10 mg/kg GW9662 every 2 days [24], totaling three injections. GW9662 was purchased from Beyotime (Shanghai, China). The IR and control groups also received the same dose of corn oil by gavage. The experimental mice were injected with pentobarbital sodium intraperitoneally and placed in the supine position. A rubber band was applied above the greater trochanter to interrupt arterial blood flow for 2 h, followed by 4 h of reperfusion to establish the IR model. Complete ischemia was characterized by acrocyanosis and cold limbs. Throughout the experiment, a laser speckle contrast imager was used to monitor lower limb blood flow (Figure 1B).

### 2.2. Bronchoalveolar Lavage Fluid (BALF)

Following ligation of the right lung hilum, the left lung was lavaged with 0.8 mL of cold PBS. The BALF underwent centrifugation at 5000 rpm for 5 min at 4°C. The supernatant was collected and preserved at −80°C for future use. Concentrations of IL-1β (Beyotime, PI301) and IL-18 (Beyotime, PI553) were measured using commercial ELISA kits following the instructions. Protein concentration in the BALF was measured using the BCA method.

### 2.3. HE and TUNEL Staining

The right lung's superior lobe was preserved in 4% paraformaldehyde, paraffin-embedded, and sectioned to 4 μm for HE and TUNEL staining. Cell apoptosis was evaluated and quantified through the TUNEL assay. The quantity of TUNEL-positive cells within each field of view was enumerated, and the apoptosis rate was computed. A double-blind design was implemented: the pathologists were unaware of the group allocation, and the technician who prepared the slides used coded labels that concealed treatment information. Five representative fields were selected from each group. Lung injury was assessed via semiquantitative scoring method by two experienced experts who were blinded to the experimental objectives.

### 2.4. Immunohistochemical (IHC)

For immunohistochemistry, lung tissue embedded in paraffin blocks was dewaxed, rehydrated, and underwent antigen retrieval, following established literature methods. The sections were subsequently blocked with primary antibodies against MPO (abcam, ab208670, 1/1000), NLRP3 (abcam, ab263899, 1/1000), IL-1β (abcam, ab283818, 1/500) and ASC (abcam, ab283684, 1/2000) overnight. The next day, the slides were stained with biotinylated secondary antibodies and visualized via DAB chromogen. The positive areas of the IHC staining were calculated via ImageJ software.

### 2.5. Western Blotting

The proteins of lung were extracted. Following the established experimental protocol [25], lung tissue was lysed in RIPA buffer with protease/phosphatase inhibitors (Beyotime, P0013B). BCA determined protein concentration. Protein concentration was determined by BCA assay, and protein expression levels were tested using WB (10% separating gel and 4% stacking gel). The primary antibodies, including anti-β-actin (abcam, ab8226, 1/10000), anti-GSDMD (abcam, ab209845, 1/1000), anti-NLRP3 (abcam, ab263899, 1/1000), anti-ASC (abcam, ab283684, 1/1000), anti-PPARγ (abcam, EPR25862-79, 1/1000), anti-AKT (abcam, ab38449, 1/2000), anti-phospho-AKT (abcam, T308, 1/1000), anti-PI3K (abcam, ab180967, 1/1000), and anti-phospho-PI3K (abcam, Y464, 1:1000) antibodies. The next day, the membranes were washed with TBS plus 0.05% Tween-20 for 30 min and incubated with secondary antibodies (1:5000) for 2 h at room temperature. The protein bands were visualized via a gel imaging system (Bio-Rad, California, USA), and the relative protein expression levels were calculated with ImageJ software (ImageJ 1.8, NIH, USA).

### 2.6. Screening of Lutein Targets

The two-dimensional structure and three-dimensional structure of lutein were obtained from MolView (http://molview.org/), as displayed in Figure 1A. Potential lutein targets were identified using the Swiss Target Prediction database and SuperPred. ALI-related genes were sourced from GeneCards (https://auth.lifemapsc.com/) and DisGeNET (https://www.disgenet.org/). A Venn diagram analysis was subsequently conducted using (http://www.ehbio.com/test/venn/#/) to identify the overlapping genes. Molecular docking between lutein and the potential protein targets was performed via AutoDock 4.0 software, with a binding energy threshold of −5.0 kcal/mol indicating potential binding affinity (Figure S2).

### 2.7. Statistical Analysis

All the data are presented as the means ± standard deviations, and the statistical analysis was performed via GraphPad Prism 5.0 (GraphPad Software Inc., San Diego, CA). Differences between groups were evaluated via one-way ANOVA and Tukey's post-hoc test. A *p* value of <0.05 was considered to indicate statistical significance. All experiments in this paper were repeated at least three times. Outlier handling and any missing data criteria were pre-specified.

## 3. Results

### 3.1. Lutein Alleviates the Lung Tissue Injury Induced by LIR

**Figure 2:**
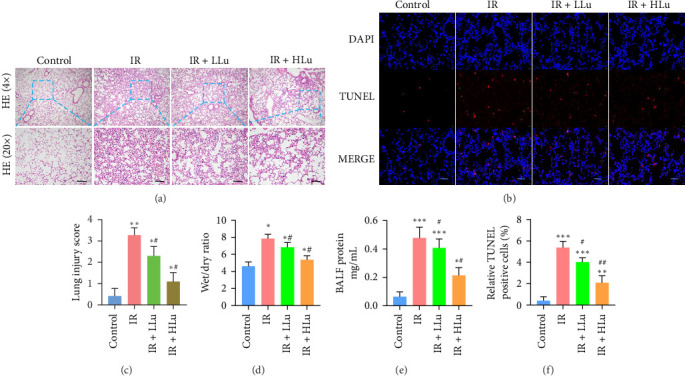
Lutein attenuated LIR-ALI. (a, c) Evaluate the pathological changes of lung tissues by HE staining (scale bars = 100 μm). (b, f) The identification of apoptotic cells was achieved through the TUNEL assay (scale bars = 50 μm). (d) The change of pulmonary edema was observed by measuring the W/D ratio. (e) The changes of the total protein levels in BALF. *⁣*^*∗*^*p* < 0.05 vs Control, *⁣*^*∗∗*^*p* < 0.01 vs Control, *⁣*^*∗∗∗*^*p* < 0.001 vs Control, ^#^*p* < 0.05 vs IR, ^##^*p* < 0.01 vs IR.

To assess lutein's impact on LIR-ALI, we examined morphological characteristics and measured the lung wet/dry (W/D) ratio and protein levels in BALF to evaluate pulmonary endothelial permeability. The IR group led to typical characteristics of ALI, including alveolar collapse, edema of the alveolar wall, and distinct interstitial thickening, as well as the infiltration of a great many neutrophils. Lutein markedly mitigated these injuries in a dose-dependent fashion (Figure 2A, C). To determine the effect of lutein on cell apoptosis in ALI, the lung tissues were evaluated using the TUNEL assay. An increased proportion of TUNEL-positive cells was observed in the IR group, with lutein remarkably decreased the percentage of TUNEL-positive cells (Figure 2B, F). Changes in the W/D ratio and total protein concentration in each experiment aligned with the histopathological analysis results when compared to the control group. Treatment with 25 mg/kg of lutein significantly decreased both the W/D ratio and total protein concentration in BALF (Figure 2D, E).

### 3.2. Lutein Alleviates Lung Inflammation Induced by LIR

**Figure 3:**
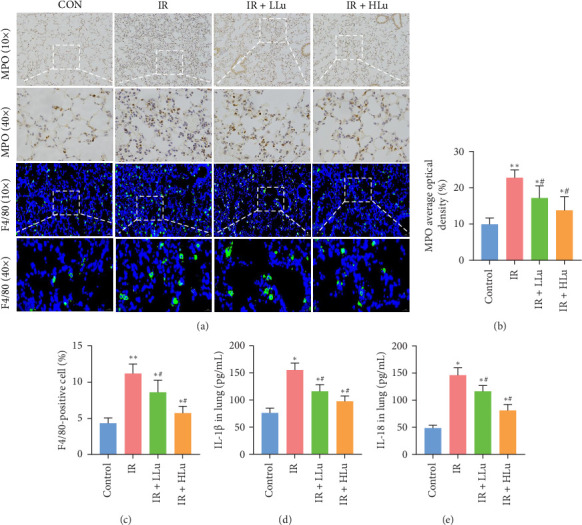
Effects of lutein on inflammation levels in LIR–ALI. (a) IHC staining of MPO and immunofluorescence staining of macrophages in the lung (scale bars = 50 μm). (b) Proportions of MPO positive cells. (c) F4/80+ cells. (d, e) Effect of lutein on IL-1β and IL-18 in lung. *⁣*^*∗*^*p* < 0.05 vs Control, *⁣*^*∗∗*^*p* < 0.01 vs Control, *⁣*^*∗∗∗*^*p* < 0.001 vs Control, ^#^*p* < 0.05 vs IR, ^##^*p* < 0.01 vs IR.

The increase in the secretion of inflammatory cells and pro-inflammatory cytokines is an important inducing factor for ALI. MPO is mainly secreted by neutrophils. MPO content alterations are linked to alveolar-capillary barrier integrity and can indirectly indicate neutrophil infiltration levels [26]. F4/80 is a marker of macrophages [27]. IHC analysis of MPO and immunofluorescent analysis of F4/80 revealed elevated proportions of MPO and F4/80 in the alveolar structures of all experimental groups compared to the control group, with the most pronounced increase observed in the IR group (Figure 3A–C). However, lutein treatment significantly inhibited the number and activity of inflammatory cells. Comparable alterations were noted in IL-1β and IL-18 within the lung. In conclusion, these findings indicate that lutein alleviates the pulmonary inflammatory response induced by limb IR (Figure 3D, E).

### 3.3. Lutein Reduced the Activation of the Inflammasome NLRP3

**Figure 4:**
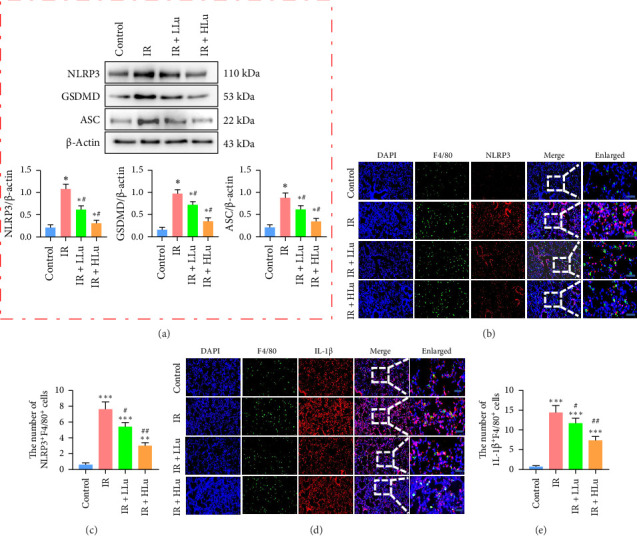
Lutein decreased the activation of the inflammasome NLRP3. (a) Western blot analysis was used to detect NLRP3, ASC, and GSDMD protein expression levels. (b, c) Immunofluorescence analysis of NLRP3 activation in macrophages. (d, e) The expression of IL-1β in macrophages was analyzed by immunofluorescence (scale bars = 50 μm). *⁣*^*∗*^*p* < 0.05 vs Control, *⁣*^*∗∗*^*p* < 0.01 vs Control, *⁣*^*∗∗∗*^*p* < 0.001 vs Control, ^#^*p* < 0.05 vs IR, ^##^*p* < 0.01 vs IR.

We employed Western blot and IHC analyses to assess the expression of NLRP3, ASC, GSDMD, and IL-1β in mouse lung tissues, aiming to determine the activation of NLRP3/GSDMD/ASC-mediated pyroptosis across different groups. ASC, GSDMD, and the NLRP3 inflammasome exhibited significant activation in the IR groups compared to the control group. However, lutein treatment mitigated these changes (Figure 4A). Macrophages are crucial elements of the lung's innate immune system. To explore whether macrophage pyroptosis is activated in LIR–ALI and exacerbates the massive release of inflammatory factors through pyroptosis, thereby promoting ALI, we used immunofluorescence colocalization techniques to stain lung tissues from the mice in each group and observed the colocalization of F4/80 with the pyroptosis-related proteins NLRP3 and IL-1β. The IR groups exhibited significant colocalization of NLRP3, IL-1β, and F4/80, with a notable rise in double-positive cells compared to the control group (Figure 4B–E). These findings further confirmed that lutein inhibits NLRP3 inflammasome-mediated macrophage pyroptosis in LIR–ALI.

### 3.4. Construction of a PPI Network of Genes Overlapping With Lutein and ALI-Related Genes

**Figure 5:**
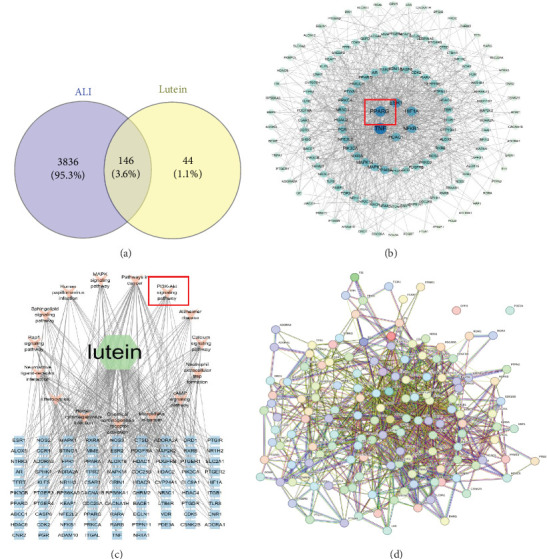
Network pharmacology prediction for lutein pre-treatment in acute lung injury. (a) Venn diagrams of lutein targets and ALI targets. (b) The PPI network of key therapeutic targets. (c) The network of drug-target-pathways was constructed. (d) PPI network exported from STRING database.

The beneficial effects of lutein on LIR-induced lung injury may involve multiple pathways and targets. A total of 190 target genes for lutein and 3982 target genes for acute lung injury were obtained through database retrieval. A Venn diagram identified 146 common targets between lutein and ALI (Figure 5A). Protein–protein interaction (PPI) data for these targets were retrieved using the STRING database.5. The genes PPAR-γ, TNF, and HIF1α, among others, are central in the PPI network, indicating their importance as targets for lutein in ALI treatment (Figure 5B, D). GO and KEGG pathway enrichment analyses were conducted using the cluster profiler package in R. The results of the GO enrichment and KEGG pathway analyses are shown in Figure S1. Network topology analysis indicates that lutein potentially influences the PI3K-AKT, TNF, MAPK, and cAMP signaling pathways by targeting TNF, HIF1α, ESR1, and PPAR-γ, thus offering therapeutic benefits for ALI (Figure 5C). The binding affinity between ligands and receptors depends on their interaction energy, with a lower energy indicating stronger binding. Molecular docking was performed between lutein and the six key targets, and lutein was able to form relatively stable complexes with each ligand molecule (Figure S2).

### 3.5. In LIR–ALI, Lutein Activates PPAR-γ, Leading to the Inhibition of the PI3K/AKT Signaling Pathway

**Figure 6:**
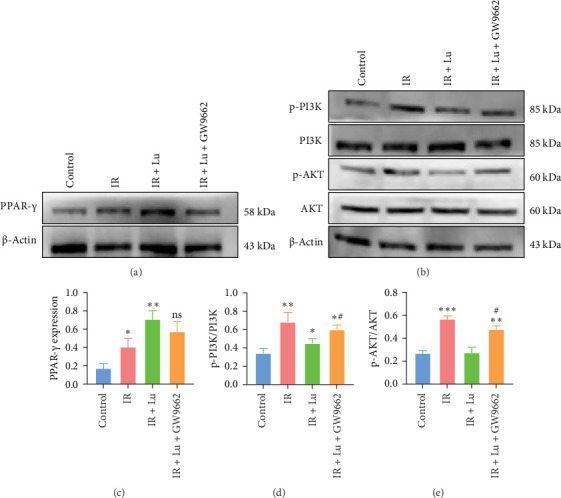
Lutein inhibited PI3K/AKT pathway by activating PPAR-γ. (a–e) The expression of PPAR-γ, p-PI3K/PI3K, and p-AKT/AKT was evaluated with western blot. *⁣*^*∗*^*p* < 0.05 vs Control, *⁣*^*∗∗*^*p* < 0.01 vs Control, *⁣*^*∗∗∗*^*p* < 0.001 vs Control, ^#^*p* < 0.05 vs IR.

Given that lutein can inhibit inflammatory responses in ALI, we aimed to identify the potential mechanisms mediating the protective effects of lutein through database network analysis. Expression levels of PPAR-γ and the PI3K/AKT pathway in lung tissue were assessed using Western blotting analysis. Compared with the control group, the injured group presented slight increases in PPAR-γ expression. Pretreatment with lutein further increases PPAR-γ expression (Figure 6). The IR group showed elevated p-PI3K/PI3K and p-AKT/AKT ratios, which were significantly decreased by lutein treatment in ALI-LIR.

### 3.6. The Therapeutic Effect of Lutein Was Reversed by the PPAR-γ Inhibitor GW9662

**Figure 7:**
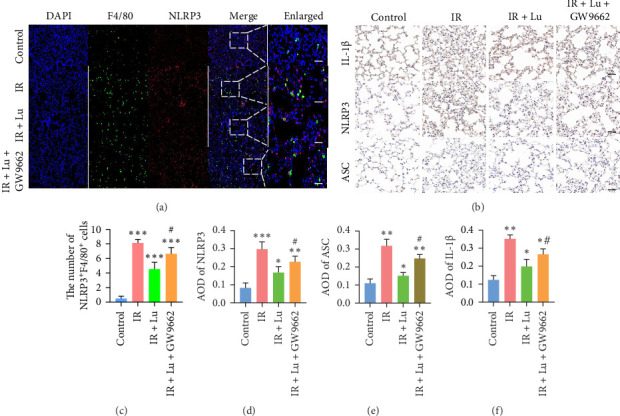
Effects of lutein on NLRP3 inflammasome-dependent signaling pathway-associated proteins in a mice model. (a) Immunofluorescence staining of NLRP3 and F4/80 in lung (scale bars = 50 μm). (b) Representative IHC images of NLRP3, ASC, and IL-1β (scale bars = 50 μm). (c–f) Semi-quantitative analysis of the positive area. *⁣*^*∗*^*p* < 0.05 vs Control, *⁣*^*∗∗*^*p* < 0.01 vs Control, *⁣*^*∗∗∗*^*p* < 0.001 vs Control, ^#^*p* < 0.05 vs IR + Lu.

To confirm the involvement of the PPAR-γ/PI3K/AKT signaling pathway in lutein treatment, the selective PPAR-γ inhibitor GW9662 was employed. Additionally, lutein reversed the increases in p-PI3K, p-AKT, NLRP3, and ASC protein levels induced by LIR–ALI, and these effects were blocked by GW9662 (Figure 7). In conclusion, these findings indicate that lutein activates PPAR-γ to inhibit the PI3K/AKT/NLRP3 pathway, thus preventing LIR–ALI.

## 4. Discussion

Prolonged limb IR may lead to pulmonary oxidative stress, inflammatory response, and pyroptosis, resulting in ALI [28]. Lutein, a carotenoid that occurs naturally, can be found in dark green leafy vegetables like kale and spinach. Research indicates that lutein possesses anti-inflammatory and immunomodulatory properties, offering significant protection against ischemia-reperfusion injury in the spinal cord, myocardium, and small intestine [29–31]. However, whether lutein has a protective effect on LIR–ALI and its underlying mechanism has not been studied yet. This study, as part of our series on LIR–ALI prevention and treatment, explored lutein's protective effects and mechanisms against LIR–ALI. The results showed that lutein significantly inhibited the pulmonary inflammatory response and pyroptosis induced by LIR. PAMPs and DAMPs stimulate neutrophils, vascular endothelial cells, and macrophages to release pro-inflammatory cytokines. The released cytokines can change the permeability of capillaries in the body, ultimately leading to pulmonary edema. LIR injury sets off a cascading inflammatory response. As a result, a vast number of inflammatory mediators are released into the bloodstream. These mediators include the interleukin family, TNF-α, ICAM, and VACM [32, 33]. The study found that lower LIR significantly elevated IL-1β and IL-18 levels in BALF, while lutein reduced these levels in a concentration-dependent manner. Lutein pretreatment also alleviated the elevation of MPO level caused by LIR. These results indicate that lutein has the efficacy of antioxidative stress and prevents the infiltration of inflammatory cells. It should be noted that the interaction between neutrophils and macrophages is extremely complex in the regulation of inflammation. Whether lutein exerts its protective effect by influencing this interaction is worthy of in-depth exploration in future studies.

Pyroptosis, a type of programmed cell death marked by the release of pro-inflammatory cytokines, plays a crucial role in lung injuries induced by diverse factors. The activation of the NLRP3 inflammasome is pivotal in the development of various lung diseases. In hyperoxia-induced lung injury, NLRP3 inflammasome activation elevates pyroptosis, contributing to bronchopulmonary dysplasia in premature infants [34]. In a mouse model of bronchopulmonary dysplasia, tert-butylhydroquinone (TBHQ), an Nrf2 activator, mitigates pyroptosis and enhances alveolar development by reducing NLRP3 inflammasome activation and oxidative stress [35]. Exposure to ambient fine particulate matter (PM2.5) also contributes to lung injury, with pyroptosis playing a crucial role. Salidroside, an active compound from *Rhodiola rosea*, protects against PM2.5-induced lung injury by inhibiting apoptosis and pyroptosis. This is achieved through the regulation of the NF-κB/NLRP3/caspase-1 signaling pathway, highlighting the potential of Salidroside as a preventative therapy for such environmental lung injuries [36]. Moreover, the role of pyroptosis in sepsis-induced lung injury has been explored, where the methylation of TTC4 and its interaction with HSP70 were found to inhibit pyroptosis in macrophages. This inhibition is crucial in reducing inflammation and lung damage in sepsis models, suggesting that targeting pyroptosis could be a viable therapeutic strategy for managing sepsis-induced lung injury [37]. These studies collectively underscore the importance of pyroptosis in the pathophysiology of lung injuries and highlight potential therapeutic interventions that target this inflammatory cell death pathway to mitigate lung damage. This study highlights the novel importance of pyroptosis in LIR-ALI, demonstrating that lutein treatment significantly decreases the expression of NLRP3, ASC, and GSDMD, as well as the secretion of IL-1β and IL-18. These findings strongly support the view that therapeutic measures aimed at manipulating pyroptosis have great potential in the treatment of ALI.

Network pharmacology analyzes the relationships among drugs, targets, and diseases by constructing network models [38]. We implemented network pharmacology to identify the relevant targets of lutein for LIR-ALI. PPI analysis indicated that PPAR-γ is the core target for treatment. KEGG analysis showed that lutein may exert beneficial effects through the PI3K/AKT signaling pathway. AKT is a downstream target of PI3K. After PI3K is activated, AKT is recruited to the plasma membrane and phosphorylated [39]. The PI3K/AKT signaling pathway plays a crucial role in pulmonary inflammation, cell survival, and oxidative stress [40]. Some studies have shown that AKT is involved in the regulation of pyroptosis [41]. By using the PPAR-γ inhibitor GW9662, we found that the inhibition of PPAR-γ contributes to the upregulation of PI3K/AKT. Consistent with our experimental results and network pharmacology analysis, we demonstrated that lutein inhibits pyroptosis in the LIR–ALI model by regulating the PPAR-γ/PI3K-AKT/NLRP3 pathway. As a natural antioxidant, lutein can scavenge excess free radicals and reduce oxidative damage, potentially aiding in post-trauma tissue repair and function recovery. Its anti-inflammatory properties may also alleviate inflammation-related complications. However, there are several limitations in the current study. Firstly, there is a lack of tissue specificity in the database. Secondly, the study only monitored pathological changes after 4 h of reperfusion, without longer monitoring; organ injury and mortality usually peak between 12 and 24 h. Therefore, early readouts may miss late pathology or survival benefits. Thirdly, our findings indicate the compound's capacity to prevent, rather than reverse, IR-induced lung damage. Whether lutein retains its efficacy when administered after the onset of injury remains to be determined, and caution should be exercised when extrapolating these results to therapeutic settings.

## 5. Conclusions

The use of lutein can effectively alleviate ALI induced by the prolonged use of a tourniquet on the lower limbs. In conclusion, the protective effect is partially mediated by regulating the PPAR-γ/PI3K-AKT/NLRP3 pathway to inhibit pyroptosis. These findings highlight lutein's dual antioxidant and antipyroptotic mechanisms, underscoring its potential as a candidate for preventing and treating LIR–ALI.

## Data Availability

The datasets utilized and analyzed in this research can be obtained upon request from the author.

## Supporting Information

Supporting Information Figure S1. KEGG pathway enrichment & GO analysis. (A) KEGG enrichment analysis. (B) GO enrichment analysis. Figure S2. Molecular docking. (A) Molecular docking analysis. (B) Molecular binding energy.
