# Supplementary material for: Lutein Alleviate Acute Lung Injury Induced by Limb Ischemia-Reperfusion Through PPAR-γ/PI3K/AKT/NLRP3 Signaling
Source: Mediators Inflamm. 2025 Nov 17;2025:2371545. doi: 10.1155/mi/2371545 (PMC12643664; doi:10.1155/mi/2371545)
Supplement: Supporting Information — Figure S1. KEGG pathway enrichment & GO analysis. (A) KEGG enrichment analysis. (B) GO enrichment analysis. Figure S2. Molecular docking. (A) Molecular docking analysis. (B) Molecular binding energy. [file 2371545.f1.doc]

**Title:** Lutein alleviate acute lung injury induced by limb ischemia-reperfusion through PPAR-γ/PI3K/AKT/NLRP3 Signaling

**Authors:** Chao Nie^a^, Zhen Liu^a^, Liang Zhang^a^, Chuanchuan Liu^a^, Hui Jiang^a^, Minghua Liu^a, *^

^a^ Department of Emergency, The First affiliated Hospital, Army Medical University, Chongqing, China

*Corresponding Author: Minghua Liu

1. mail address: [minghua_liu@tmmu.edu.cn](mailto:minghua_liu@tmmu.edu.cn)
2. **Table of Contents**

Fig. S1. KEGG pathway enrichment & GO analysis I

Fig. S2.Molecular docking II


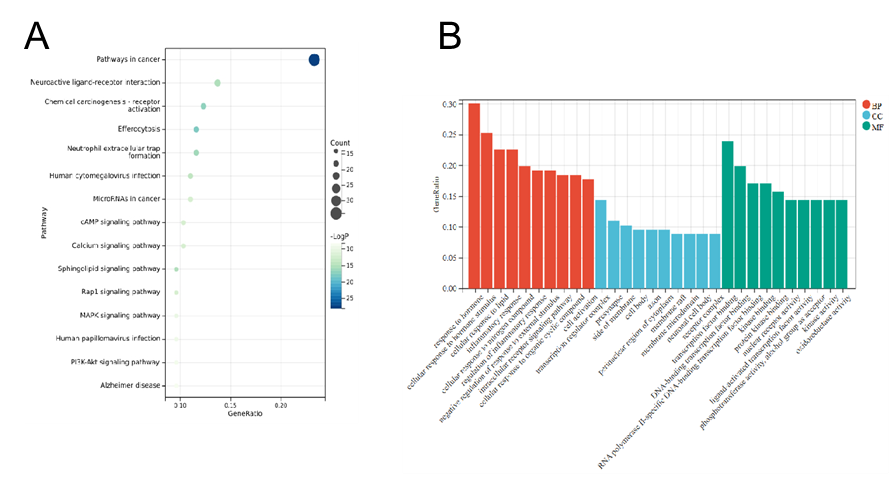


Fig S1. KEGG pathway enrichment & GO analysis.(A) KEGG enrichment analysis. (B) GO enrichment analysis.


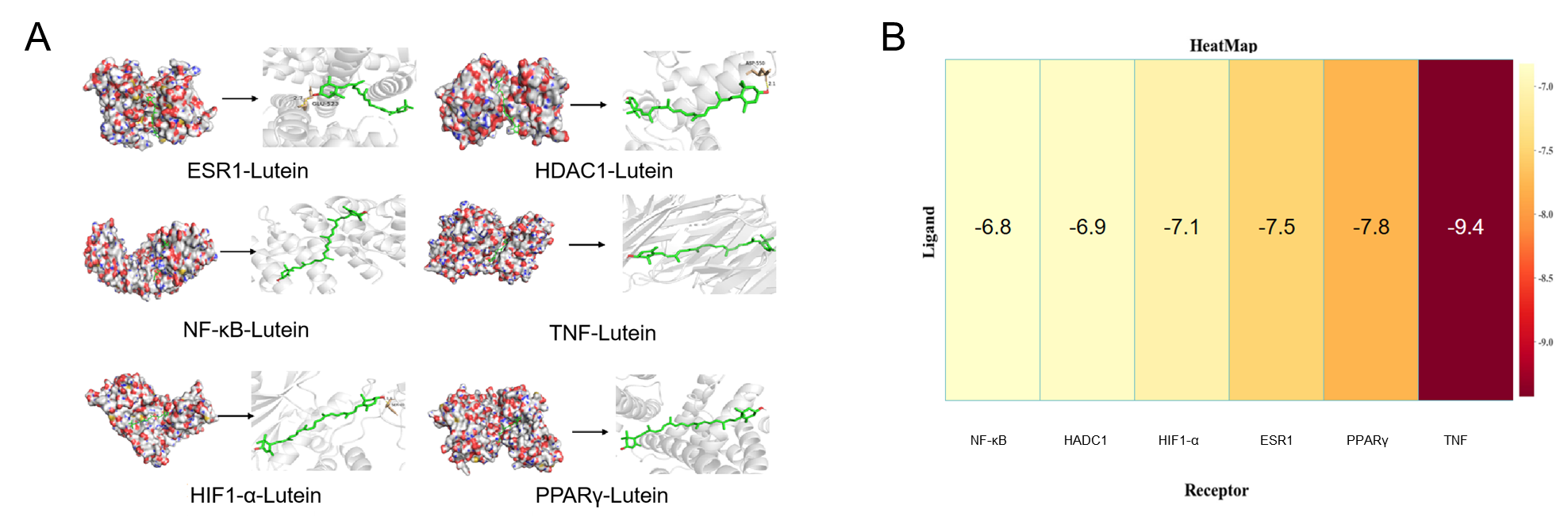


Fig S2. Molecular docking. (A) Molecular docking analysis. (B) Molecular binding energy.
